# Aurora kinase B dependent phosphorylation of 53BP1 is required for resolving merotelic kinetochore-microtubule attachment errors during mitosis

**DOI:** 10.18632/oncotarget.16225

**Published:** 2017-03-15

**Authors:** Haibo Wang, Bin Peng, Raj K. Pandita, David A. Engler, Risë K. Matsunami, Xingzhi Xu, Pavana M. Hegde, Brian E. Butler, Tej K. Pandita, Sankar Mitra, Bo Xu, Muralidhar L. Hegde

**Affiliations:** ^1^ Department of Radiation Oncology, Houston Methodist Research Institute, Houston, TX, USA; ^2^ Houston Methodist Neurological Institute, Houston, TX, USA; ^3^ Beijing Key Laboratory of DNA Damage Response and College of Life Science, Capital Normal University, Beijing, China; ^4^ Proteomics Programmatic Core Laboratory, Houston Methodist Research Institute, Houston, TX, USA; ^5^ Weill Medical College of Cornell University, New York, NY, USA; ^6^ Department of Oncology, Southern Research Institute, Birmingham, AL, USA

**Keywords:** 53BP1, aneuploidy, chromosome segregation, merotelic attachment

## Abstract

Defects in resolving kinetochore-microtubule attachment mistakes during mitosis is linked to chromosome instability associated with carcinogenesis as well as resistance to cancer therapy. Here we report for the first time that tumor suppressor p53-binding protein 1 (53BP1) is phosphorylated at serine 1342 (S1342) by Aurora kinase B both in vitro and in human cells, which is required for optimal recruitment of 53BP1 at kinetochores. Furthermore, 53BP1 staining normally localized on the outer kinetochore, extended to the whole kinetochore when it is merotelically-attached, in concert with mitotic centromere-associated kinesin. Kinetochore-binding of pS1342-53BP1 is essential for efficient resolving of merotelic attachment, a spontaneous kinetochore-microtubule connection error that usually causes aneuploidy. Consistently, loss of 53BP1 results in significant increase in lagging chromosome events, micronuclei formation and aneuploidy, due to the unresolved merotely in both cancer and primary cells, which is prevented by ectopic wild type 53BP1 but not by the nonphophorylable S1342A mutant. We thus document a novel DNA damage-independent function of 53BP1 in maintaining faithful chromosome segregation during mitosis.

## INTRODUCTION

53BP1 has multiple roles in mammalian DNA damage response (DDR), specifically for DNA double-strand break (DSB) repair via non-homologous end-joining (NHEJ) and has been implicated in the activation of intra-S-phase and G2/M checkpoints [1–8]. 53BP1 is recruited at DSB sites in Ataxia Telangiectasia Mutated (ATM)-dependent manner, following histone H2AX phosphorylation (γ-H2AX), recruitment of MDC1, RNF8 and RNF168 [9]. When 53BP1 was first observed to accumulate at DSB sites, its specific localization on outer kinetochores and its hyper-phosphorylation during mitosis were also reported [10]. Interestingly, 53BP1 tightly binds to kinetochores during mitosis but is excluded at DNA damage sites [10–12]. Recent studies have demonstrated that the exclusion of 53BP1 from mitotic DSB sites requires its phosphorylation at T1609 and S1618 residues within the ubiquitination-dependent recruitment (UDR) motif by CDK1 and PLK1 [13, 14]. While mitotic inhibition of DSB repair may have evolved to protect chromosomes against telomere fusion [14], its inhibition also renders mitotic cells hypersensitive to ionizing radiation (IR). However, the kinetochore binding of 53BP1 in unstressed mitotic cells indicates its DNA damage-independent mitotic function, which has never been investigated. Furthermore, 53BP1 null mouse cells display aneu- and tetraploidy [4, 15], suggesting that 53BP1 may be involved in the regulation of kinetochore-microtubule attachment and chromosome segregation.

Here, we report that 53BP1 is phosphorylated by Aurora kinase B at serine 1342 (S1342), and the phosphorylation is critical for 53BP1 recruitment to kinetochores. In addition, 53BP1 is distributed at merotelically attached kinetochores and co-localized with distorted anti-centromere antibody (ACA) or HEC1 staining, markers for merotelic attachment [16, 17]. Merotely is a spontaneous kinetochore-microtubule attachment error that occurs when a kinetochore is connected by microtubule bundles from opposite spindle poles during mitosis, and is a major cause of aneuploidy in mammalian cells [18, 19]. 53BP1 depletion or loss of its Aurora B dependent phosphorylation site S1342 in human cells significantly enhances merotelic attachment, lagging chromosome and aneuploidy. These findings imply a novel DNA damage-independent function of 53BP1 linked to Aurora B-mediated S1342 phosphorylation during mitosis to protect against aberrant chromosomal segregation.

## RESULTS

### Aurora B phosphorylates 53BP1 at residue S1342 during mitosis, which is required for optimal recruitment of 53BP1 at kinetochores

**Figure 1:**
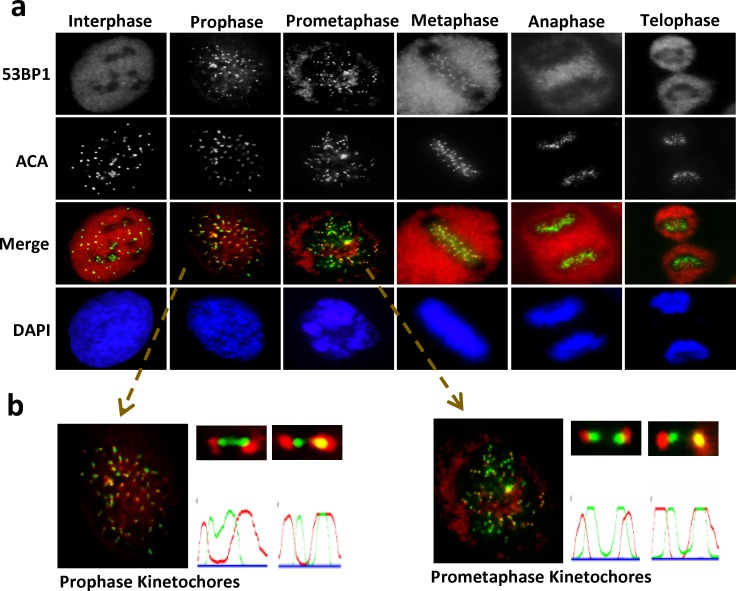
Unique recruitment pattern of 53BP1 at kinetochore during mitosis **a**. Unsynchronized U2OS cells were plated on cover slides and processed for IF with a 53BP1 antibody (red) and anti-centromere antibody (ACA, green). Nuclei were stained by DAPI (blue). **b**. Selected foci were enlarged for visualization. Quantitation of fluorescent intensity using MetaMorph software obtained by drawing a 2-pixel-width line through the kinetochores.

To elucidate the functional implications of 53BP1's localization at kinetochores during mitosis [10, 12], we analyzed its recruitment pattern at different mitotic stages by immunofluorescence (IF) microscopy. Endogenous 53BP1 appears on kinetochores (visualized by ACA) at prophase, is partially retained until metaphase and then dissociates during anaphase (Figure 1a). A similar localization pattern was observed in cells expressing ectopic FLAG-53BP1 (Supplementary Figure S1), consistent with the report of 53BP1's co-localization with CENP-E on outer kinetochores during mitosis [10]. In addition, we observed increased 53BP1 staining in the cytoplasm especially in metaphase and anaphase (Figure 1a), which is likely due to its release from the kinetochores [10].

**Figure 2:**
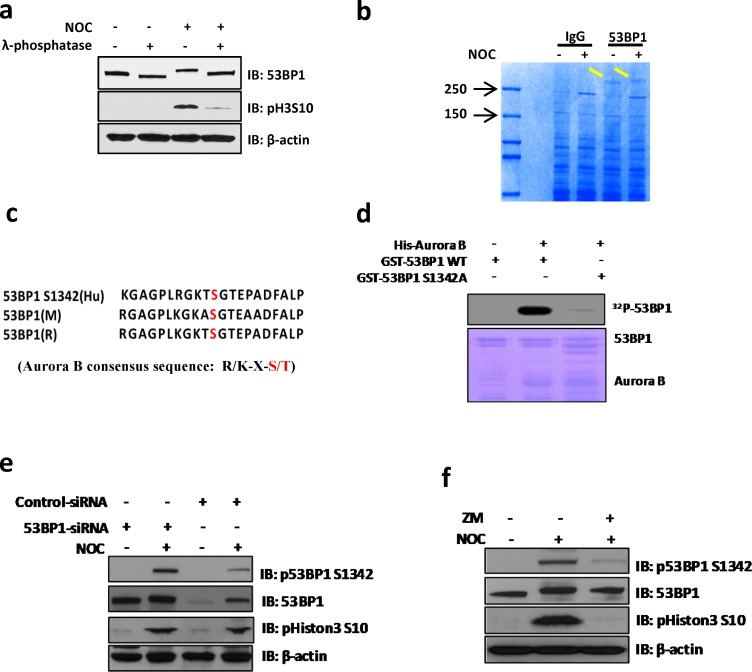
Aurora B phosphorylates 53BP1 at residue S1342 during mitosis **a**. Western blotting shows that endogenous 53BP1 are hyper-phosphorylated in NOC treated U2OS cells but dephosphorylated by treatment with λ-phosphatase. The phospho Histon3 S10 indicate mitotic arrest in response to NOC treatment. **b**. Endogenous 53BP1 was immunoprecipated from unsynchronized or NOC-treated cells with 53BP1 antibody. The resulting 53BP1 bands (arrows) were Coomassie-stained on a SDS-PAGE gel. **c**. The S1342 residue is conserved in human, mouse, and rat 53BP1 within the Aurora B kinase motif as determined by sequence alignment analysis (red indicates the position of S1342 in short sequences). The Aurora B consensus sequence is shown (red indicates serine or threonine in the motif). **d**. An *in vitro* kinase assay was performed by incubating purified His-Aurora B with WT or mutant GST-53BP1 (S1342A) in the presence of ^32^P-ATP. **e**. SiRNAs transfected U2OS cells were treated with NOC for 14 h followed by immunoblotting with indicated antibodies. Phospho signal of S1342 is significantly decreased in 53BP1 depleted cells. **f**. Immunoblotting of mock or NOC-synchronized cell extracts. For Aurora inhibitor ZM-treated sample, cells were treated with NOC plus ZM for 8 h, followed by mitotic cell harvest by shake-off.

The unique localization pattern of 53BP1 at the kinetochore during specific mitotic stages suggests its involvement in chromosome segregation. Although most 53BP1 staining was excluded from or only partially co-localized with kinetochore pairs, a small fraction of 53BP1 completely co-localized with one of the kinetochores (Figure 1b). This dynamic association of 53BP1 with kinetochores during mitotic progression in unstressed cells led us to further investigate its DNA damage-independent functions. To explore this possibility, we synchronized U2OS cells with nocodazole (NOC) and collected mitotic cells by mechanical shake-off. Immunoblotting of whole cell extracts showed significant mobility shift for endogenous as well as ectopic 53BP1 in NOC-treated, but not in control cells (Figure 2a and Supplementary Figure S2a). The mobility shift disappeared after the cell extracts were treated with a λ-protein phosphatase, indicating that the slower band represented phosphorylated 53BP1. We co-immunoprecipitated (co-IP'd) endogenous 53BP1 from unsynchronized or NOC-synchronized U2OS cells. Coomassie-staining of SDS-PAGE gels revealed bands corresponding to unmodified 53BP1 in unsynchronized cells, but a predominant low mobility band representing phosphorylated 53BP1 was visualized in co-IP from mitotic cells (Figure 2b). Mass spectroscopy of excised bands, followed by comprehensive bioinformatics analysis, identified a phosphorylation site S1342 in 53BP1, highly conserved among the human and rodents (Figure 2c). Further characterization of the kinase-binding motifs in 53BP1 showed that S1342 is localized within the classic consensus motif [R/K]X[S/T] of the chromosome passenger kinase protein, Aurora B (Figure 2c) [20, 21]. To confirm that 53BP1 is phosphorylated by Aurora B, we generated bacterial expression plasmids for His-tag Aurora B and glutathione S-transferase (GST)-tag 53BP1 polypeptides (aa 1300-1750) containing S1342 corresponding to the wild-type (WT) sequence or a mutated residue Ser1342Ala (S1342A). Synthetic peptides were used as substrates in *in vitro* kinase assay using γ^32^P-ATP. Strong radioactivity, indicating phosphorylation, was detected in the WT GST-53BP1 peptide, but not the S1342A-containing peptide in the presence of His-Aurora B (Figure 2d and Supplementary Figure S2b). We next examined *in cellulo* phosphorylation of 53BP1 by obtaining a custom-generated antibody specific to phosphorylated 53BP1 at S1342 (p-S1342-53BP1). This exhibited >1000-fold higher sensitivity for p-S1342-containing 53BP1 over the non-phosphorylated control with a blocking peptide *in vitro* (Supplementary Figure S2c). Specific immunoreactivity with the p-S1342-53BP1 antibody was observed in NOC-treated HeLa and U2OS cell extracts compared to mock-treated cell extracts (Supplementary Figure S2d). Furthermore, the p-S1342-53BP1 signal dramatically decreased in 53BP1-depleted mitotic cells (Figure 2e), confirming that the antibody specifically recognized p-S1342 53BP1. To conform the Aurora B's specific role in phosphorylating 53BP1 at S1342 *in cellulo*. U2OS cells were treated with Aurora B kinase inhibitor, ZM447439 (ZM), which selectively blocks Aurora B kinase activity without affecting its kinetochore localization and slightly impacts NOC-induced SAC activation [22, 23]. This allowed collection of NOC-synchronized mitotic cells in the absence of Aurora B kinase activity. Lack of a p-S1342-53BP1 band after immunoblotting of ZM-treated mitotic cell extracts (Figure 2f) confirmed that Aurora B specifically phosphorylates 53BP1 at S1342. These results clearly demonstrated that Aurora B phosphorylates 53BP1 at S1342 both in vitro and in human cells.

**Figure 3:**
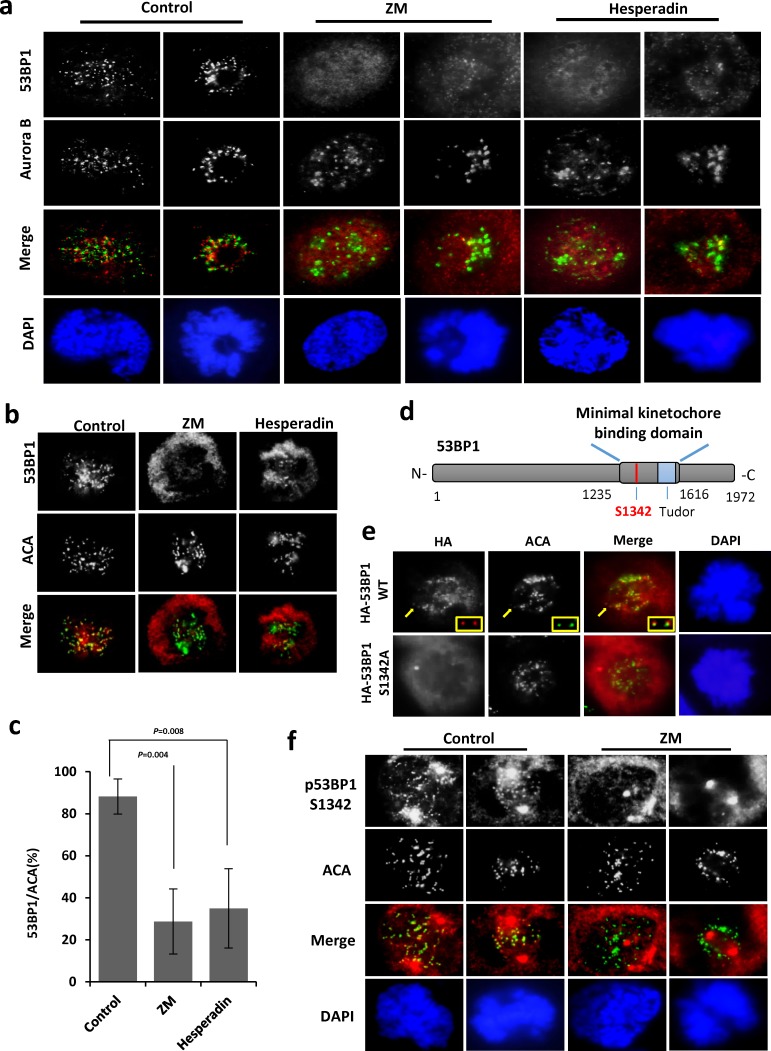
Aurora B dependent phosphorylation is required for optimal recruitment of 53BP1 at kinetochores **a**. Co-localization of 53BP1 with Aurora B (green) in mock-, ZM- or Hesperadin-treated mitotic cells. **b**. Co-localization of 53BP1 with ACA (green) in mock-, ZM- or Hesperadin-treated mitotic cells. **c**. Quantitation of 53BP1/ACA co-localization. The data are expressed as mean ± SD. **d**. Schematic of the location of S1342 in the 53BP1 KBD. **e**. IF with anti-HA antibody and ACA (green) following transient transfection of HA-53BP1 WT or HA-53BP1 S1342A into 53BP1 depleted U2OS cells. Insets indicate enlarged representations of co-localized HA-53BP1 and ACA in transfected cells. **f**. IF showing that p-S1342-53BP1 is absent from kinetochores (ACA labeled in green) in ZM-treated mitotic cells.

During cell division, Aurora B recruits a large number of proteins to subcellular compartments, including kinetochores and the midbody, via direct interaction and/or by specific phosphorylation. To address if kinetochore localization of 53BP1 relies on Aurora B kinase activity, we first tested if 53BP1 stably associates with Aurora B *in situ* using proximity ligation assay (PLA). In this assay, interaction of two proteins in close proximity is visualized as PLA foci [24, 25]. A significant number of PLA foci were observed for the 53BP1-Aurora B and p-S1342-53BP1-Aurora B complexes only in pro-metaphase but not in interphase cells (Supplementary Figure S2e), although the Aurora B signal was not detected in immunoblotting of 53BP1 co-IP (Supplementary Figure S3a), likely due to the transient nature of their association. While IF analysis in control- vs. Aurora B inhibitor ZM-treated U2OS cells showed lack of 53BP1's localization on kinetochores, particularly in prophase (Figure 3a), although few 53BP1 foci were still detectable during pro-metaphase, both the number and staining intensity of the foci were markedly decreased in ZM-treated cells, while kinetochore localization of Aurora B was not affected. Similar pattern were observed after treating cells with another commonly used Aurora B inhibitor, Hesperadin [26] (Figure 3a). We also analyzed co-localization of 53BP1 with ACA in ZM- and Hesperadin-treated cells. Figure 3b and 3c show significant decrease in 53BP1 kinetochore localization in cells after inhibition of Aurora B kinase.

A minimal kinetochore-binding domain (KBD, aa1220-1601) was previously mapped in murine 53BP1 (corresponding to aa 1235-1616 in human 53BP1), which also includes the minimal region required for irradiation-induced DNA damage focus formation [10, 27]. Because the S1342 residue lies within the KBD (Figure 3d), and in view of Aurora B-dependent recruitment of 53BP1 to kinetochores (Figure 3a, 3b and 3c), we considered if S1342 phosphorylation is necessary for the optimal binding of 53BP1 to the kinetochore. We generated an N-terminal HA tagged expression vector for aa 1235-1616 of human 53BP1 (HA-53BP1-KBD), with and without the S1342A mutation (HA-53BP1-KBD-S1342A), and transfected U2OS cells with the plasmids to express WT and mutant KBD at comparable levels (Supplementary Figure S3b). IF studies showed that HA-53BP1-KBD, but not S1342A, co-localized with ACA on the kinetochore (Supplementary Figure S3c). Consistently, full length HA-53BP1 WT but not the S1342A mutant that we later constructed showed clear kinetochore staining in U2OS cells (Figure 3e and Supplementary Figure S4). In addition, we also performed IF with p-S1342-53BP1 antibody and observed kinetochore staining of p-S1342-53BP1 in control but not in ZM-treated cells (Figure 3f). These results show that Aurora B-mediated S1342 phosphorylation regulates 53BP1 recruitment at kinetochores during mitosis.

### Phosphorylation of 53BP1 at S1342 by Aurora B is not involved in its DDR functions

**Figure 4:**
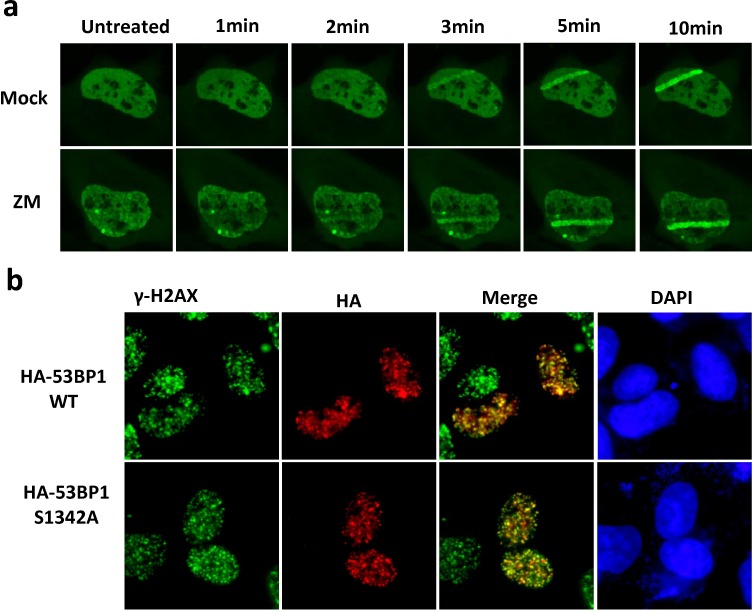

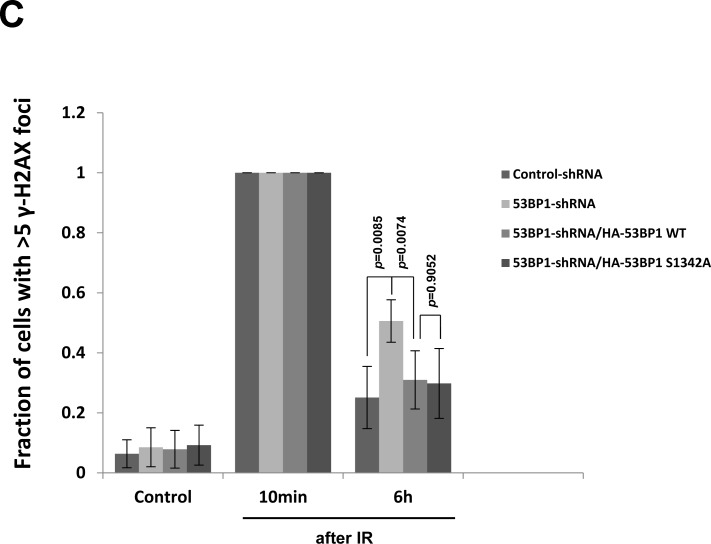
Phosphorylation of 53BP1 at S1342A by Aurora B is not involved in DNA repair signaling **a**. Immunofluorescence showing recruitment of GFP-53BP1 at laser induced DNA damage tracks in either mock or Aurora kinase B inhibitor ZM treated cells. **b**. Immunofluorescence showing that both HA-53BP1WT and HA-53BP1 S1342A form irradiation induced foci, which are co-localized with γ-H2AX (green). **c**. Quantitation of the number of γ-H2AX foci in control shRNA, 53BP1 shRNA, 53BP1 shRNA/HA-53BP1 WT and 53BP1 shRNA/HA-53BP1 S1342A co-transfected cells, before and after irradiation.

Because 53BP1 is a key protein in the DSB repair in mammalian cells, we further examined if phosphorylation of 53BP1 at S1342 is required for its DDR function. In response to DSB damage, 53BP1 binds to the DSB site and acts as scaffold to recruit additional regulators including RIF1 and PTIP [28]. We first analyzed recruitment of 53BP1 at the UV-laser induced DNA damage tracks after treatment with Aurora B inhibitor. Figure 4a showed that Aurora B kinase activity is not required for 53BP1's accumulation at DNA damage tracks. We also tested ionizing radiation induced foci formation of WT 53BP1 or the S1342A mutant. As shown in Figure 4b, HA-53BP1 S1342A is able to form foci that co-localized with γ-H2AX after irradiation, similar to that observed with WT HA-53BP1. Next, we examined if the expression of 53BP1-S1342A mutant impacts DSB repair by monitoring the kinetics of γ-H2AX foci disappearance. 48 h after co-transfection with various plasmids, DSBs were induced by ionizing radiation (2 Gy) and γ-H2AX foci were counted at indicated intervals. Most DSBs could be repaired in control cells 6 hours after irradiation but not in 53BP1 knockdown cells. However, both HA-53BP1 WT and HA-53BP1 S1342A rescued the DSB repair at comparable levels in endogenous 53BP1 depleted cells (Figure 4c). Together, these observations indicate that the phosphorylation of 53BP1 at S1342 by Aurora kinase B is not involved in DSB repair signaling.

### Loss of 53BP1 results in lagging chromosome formation

**Figure 5:**
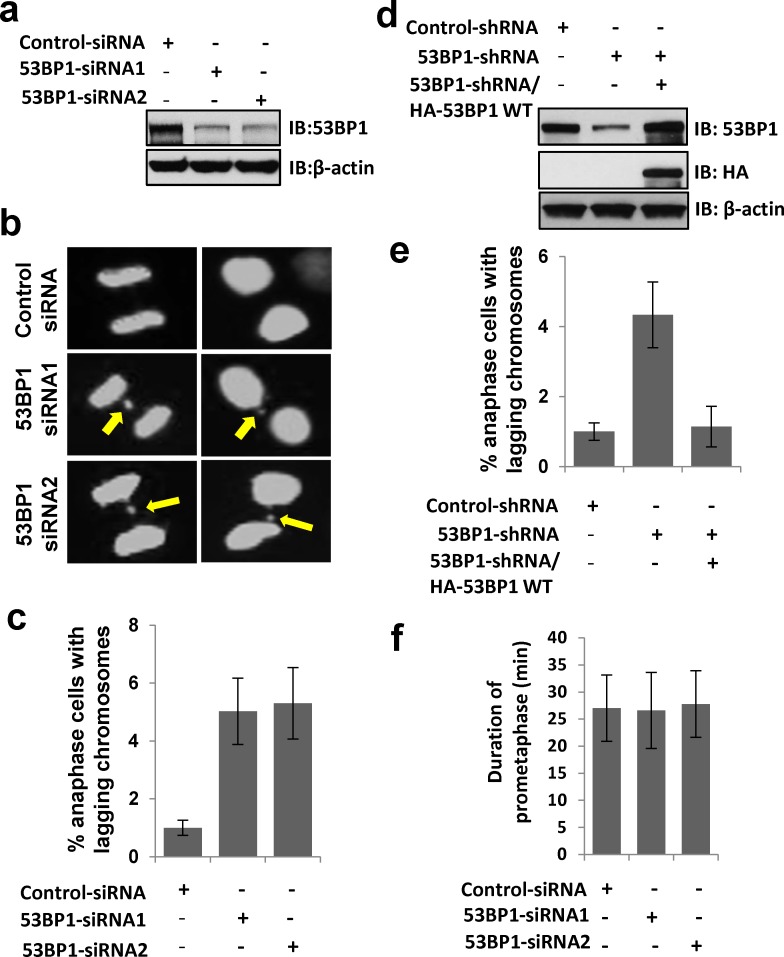
53BP1 loss results in lagging chromosome formation **a**. Immunoblotting reveals >80% depletion 53BP1 by two independent siRNA oligomer transfections in U2OS cells. β-actin served as the loading control. **b**. Time-lapse microscopy of U2OS cells stably expressing EGFP-H2B following transient transfection with control or two independent 53BP1 siRNA oligomers. Arrows indicate lagging chromosomes. **c**. Quantitation of increased lagging chromosomes in 53BP1 depleted cells in (b). Data are expressed as mean ± SD. **d**. Immunoblotting reveals >80% depletion of 53BP1 by 3′UTR-specific shRNA, which allowed ectopic expression of HA-53BP1. **e**. Ectopic FLAG-53BP1 rescues lagging chromosome events in 53BP1-depleted cells measured by time-lapse microscopy. **f**. Duration of pro-metaphase in control and 53BP1-depleted cells measured by time-lapse microscopy. The data are expressed as mean ± SD.

To further explore the mitotic function of 53BP1 as a kinetochore binding protein, we generated a U2OS cell line stably expressing EGFP-histone-H2B, which allows visualization of chromosome dynamics and mitotic timing by time-lapse microscopy. Impact of 53BP1 depletion on mitosis was tested using two independent siRNA oligomers that caused >80% depletion of 53BP1 in the EGFP-H2B stable cell line (Figure 5a). Time-lapse microscopy showed significant accumulation of lagging chromosomes in 53BP1-depleted anaphase cells (Figure 5b and 5c), which was rescued by the ectopic WT 53BP1 expression (Figure 5d and 5e). However, 53BP1 depletion had no effect on nuclear morphology along with normal chromatin condensation in prophase, sister chromatid alignment in metaphase and chromosome segregation timing in anaphase. In addition to lagging chromosome events, 53BP1-depleted cells also showed deficiency in abscising or resolving spontaneously formed chromosome bridges (unpublished data), which indicates a potential role of 53BP1 in protecting against tetraploidy.

Mammalian cells activate the spindle assembly checkpoint (SAC) in response to unconnected (premature) kinetochores during early mitosis, as a protective response against lagging chromosomes. Activated SAC causes mitotic arrest during pro-metaphase to facilitate the correction of kinetochore connection errors and restore bipolar spindles [29–31]. We observed no detectable change in pro-metaphase duration of 53BP1-depleted cells (Figure 5f). This strongly suggests that 53BP1 is not required for normal SAC function, consistent with an earlier study [32], and suggested an alternative mechanism of 53BP1's role in preventing chromosome lagging phenotype.

### 53BP1 is required for resolving merotelic attachments during mitosis

**Figure 6:**
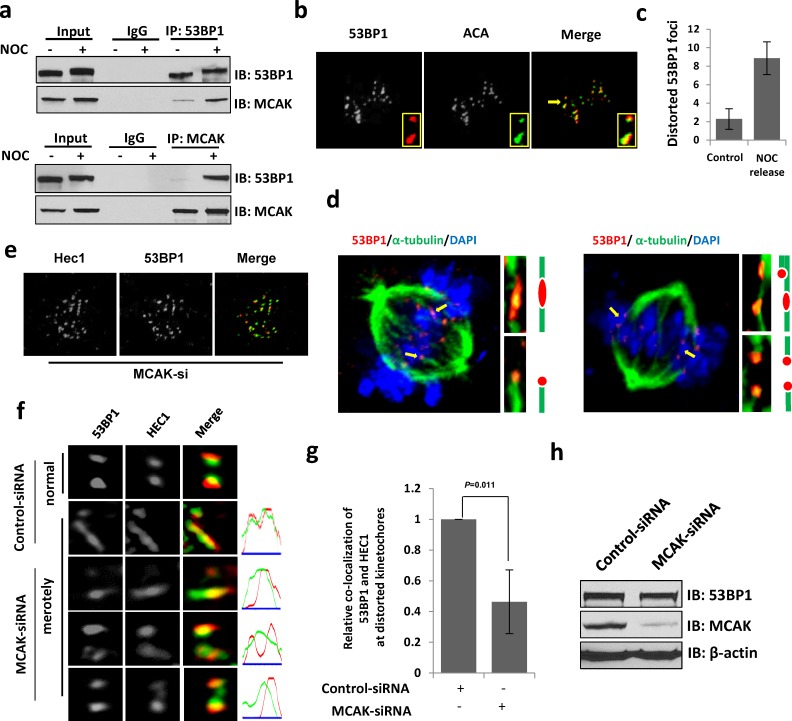
53BP1 is required for resolving merotelic attachments during mitosis **a**. Endogenous immunoprecipitation of 53BP1 or MCAK from extracts of unsynchronized or NOC-synchronized mitotic cells. **b**. IF with 53BP1 antibody and ACA (green). Insets indicate enlarged representations of co-localized 53BP1 and ACA on normal and distorted kinetochores. **c**. Quantitation of distorted 53BP1 foci in prometaphase cells. **d**. IF with 53BP1 antibody and α-tubulin antibody (green). Chromosomes were stained by DAPI (blue). Magnified images show merotelic attachments (upper) and monopolar attachments (lower) of 53BP1 by microtubule bundles. **e**. IF with 53BP1 antibody and HEC1 antibody (green) in MCAK-depleted cells. **f**. 53BP1 and HEC1 (green) staining at distorted kinetochores in control or MCAK depleted cells. **g**. Quantitation of relative co-location of 53BP1 and HEC1 or format distorted kinetochores in (f). Data are expressed as mean ± SD. **h**. Immunoblotting reveals that the protein level of 53BP1 is not affected by the down regulation of MCAK.

To determine how 53BP1 depletion causes chromosome lagging, we analyzed the 53BP1 co-IP complex from human cells after nocodazole (NOC) synchronization and identified the mitotic-centromere-associated kinesin (MCAK) protein by mass spectroscopy screening. Immunoblotting of 53BP1 co-IP and a reverse MCAK co-IP further confirmed their mitosis-dependent *in-cell* association (Figure 6a). MCAK, a microtubule depolymerase plays a critical role in ensuring accurate chromosome segregation by depolymerizing improperly attached microtubules at the kinetochore [16, 33–37]. Based on the observation that 53BP1 localizes at pre-anaphase kinetochores and interacts with MCAK, as well as the accumulation of lagging chromosomes in 53BP1-depleted cells, we hypothesized that 53BP1 is involved in merotelic kinetochore orientation. Merotelic attachments spontaneously occur in mitotic cells in about 1.2% of normal mammalian cell population, but at a higher rate in cancer cells [18, 19]. These are typically identified by microscopy based on distorted foci of merotelically-attached kinetochores and kinetochore-binding proteins. Such distortions are presumably caused by unequal pulling forces from opposite spindle poles due to improper microtubule connection and distorted ACA staining has been used as an indicator of merotelically-attached kinetochores [17]. Using confocal microscopy we detected a strong co-localization of 53BP1 with ACA on distorted or stretched kinetochores (Figure 6b). Interestingly, the 53BP1 signal was mostly excluded from ACA staining of normal kinetochores but extended and completely co-localized with ACA at distorted kinetochores. This observation may indicate that 53BP1 is dynamically extended to the inner kinetochore, likely due to its stretching by microtubules. Previous studies have shown increased merotelic attachments after release from NOC treatment [19, 38]. Hence, after NOC treatment, we released the cells from mitotic block for 10 min to allow re-connection of microtubules and kinetochores in order to attribute the distorted 53BP1 foci to merotelic attachment. We observed a marked increase in distorted 53BP1 and ACA foci in response to NOC treatment compared to mock-treated cells (Figure 6c). Merotely typically involves a single kinetochore connected by microtubules from opposite spindle poles. Following up our observation that 53BP1 enriches at merotelically attached kinetochores, we co-stained 53BP1 with α-tubulin to confirm the merotelic attachment of 53BP1. Figure 6d clearly shows 53BP1's staining distributed at merotelically-attached kinetochores by opposite microtubule bundles with an average incidence of ∼3.4% (7 merotelically attached kinetochore-53BP1 staining were identified in 206 kinetochores from 21 mitotic cells). We thus believe that 53BP1 normally localizing on outer kinetochores, completely covers merotelically-attached kinetochores.

Next, we tested the role of MCAK in 53BP1 association with kinetochores or merotelic attachments. Here we used kinetochore protein HEC1, another established marker for merotelically attached kinetochore [16]. Although MCAK knockdown did not influence the binding of 53BP1 to kinetochores (Figure 6e), distribution of 53BP1 at merotelically attached kinetochores greatly decreased (Figure 6f and 6g). However, the 53BP1 level was unchanged in MCAK-depleted cells (Figure 6h). This suggests that MCAK may stabilize 53BP1 on merotelically-attached kinetochores during mitosis.

**Figure 7:**
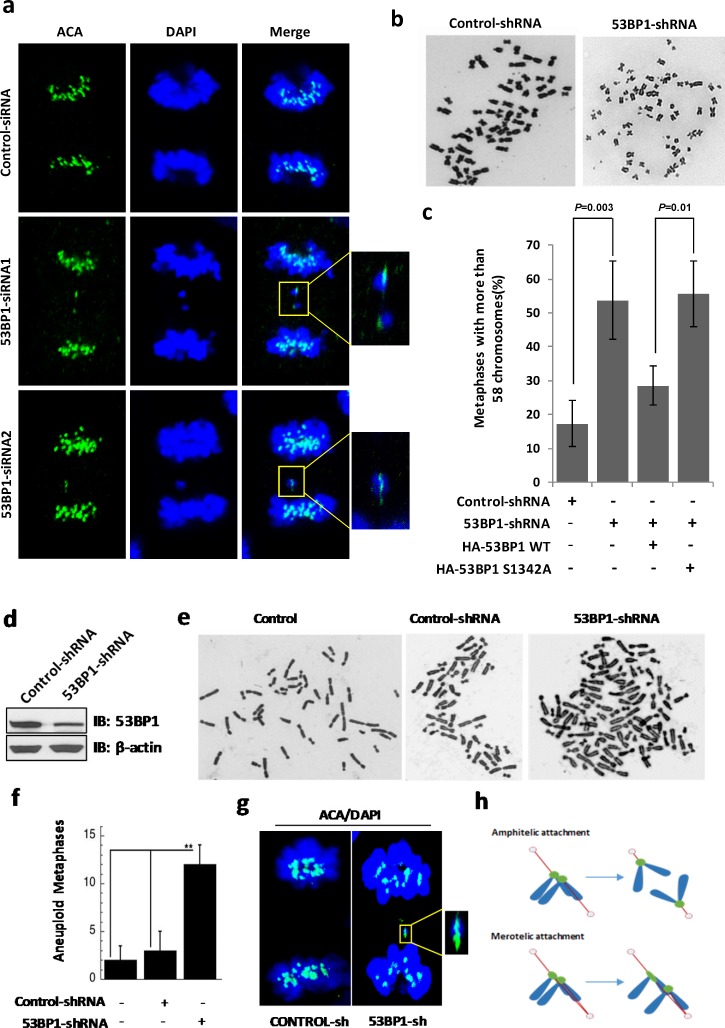
53BP-1 depletion causes chromosome segregation errors and aneuploidy in U2OS (a-c) and BJ-hTERT (d-g) cells **a**. IF with ACA antibody (green). Chromosomes were stained by DAPI (blue). Fifteen anaphase cells each from the control group and from two independent 53BP1 siRNA-transfected cell subgroups were analyzed. The two 53BP1 siRNA subgroups showed three and four cells with stretched kinetochores, respectively, but no stretched kinetochores were observed in control cells. Insets indicate paired kinetochores and distorted kinetochores. **b**. Giemsa-stained chromosomes from metaphase spreads of control and 53BP1 depleted U2OS cells. **c**. Quantitation of cells with over 58 chromosomes in control shRNA, 53BP1 shRNA, 53BP1 shRNA/HA-53BP1 WT and 53BP1 shRNA/HA-53BP1 S1342A co-transfected U2OS cells. Data are expressed as mean ± SD (**p < 0.05). **d**. Immunoblotting reveals depletion of 53BP1 by shRNA transfection in BJ-hTERT cells. β-actin served as the loading control. **e**. Giemsa-stained chromosomes from metaphase spreads of untransfected, control shRNA or 53BP1 shRNA transfected BJ-hTERT cells. **f**. Quantitation of chromosome numbers other than 46 in untransfected, control shRNA and 53BP1 shRNA transfected BJ-hTERT cells. Data are expressed as mean ± SD (**p < 0.05). **g**. IF with ACA antibody (green). Chromosomes were stained with DAPI (blue). Fifteen anaphase cells each from the control and 53BP1 shRNA-transfected cell subgroups were analyzed. The 53BP1 shRNA subgroups showed four cells with stretched kinetochores but no stretched kinetochores were observed in control cells. Insets represent distorted kinetochores. **h**. Schematic diagram of the generation of distorted kinetochores in anaphase.

Due to the enrichment of 53BP1 at merotelic kinetochores and increased lagging chromosome events in 53BP1-depleted cells, we tested if 53BP1 is involved in merotely correction. Given their connection to both spindle poles, kinetochores of lagging chromosomes formed due to unresolved merotely are highly stretched during anaphase [17]. We performed IF with control or 53BP1 siRNA transfected cells. As expected, lagging chromosomes with highly distorted kinetochores were consistently observed in 53BP1-depleted anaphase cells (Figure 7a). As previously demonstrated, merotelic attachment is a major cause of aneuploidy in mammalian cells [19]. We next examined the phenotype of aneuploidy in 53BP1-depleted cells to further confirm the requirement of 53BP1 for merotely correction. Giemsa staining of metaphase chromosome spreads demonstrated elevated aneuploidy in 53BP1 depleted cells compared to the control U2OS cells (Figure 7b and 7c). As most cancer cells including U2OS are highly aneuploid to begin with, we further tested the effect of 53BP1 loss in immortalized human fibroblast line BJ-hTERT (human telomerase reverse transcriptase) cells, which are diploid with 46 chromosomes. Endogenous 53BP1 was ∼70% down regulated by 53BP1 shRNA in BJ-hTERT cells (Figure 7d), which resulted in significant increase in aneuploidy (Figure 7e and 7f). Consistently, we also observed distorted kinetochores in anaphase in 53BP1 depleted BJ-hTERT cells (Figure 7g). A schematic diagram for the generation of distorted kinetochores in anaphase is represented in Figure 7h. Together, these data support our conclusion that 53BP1 prevents aneuploidy in human cells by resolving spontaneous errors, merotelic kinetochore-microtubule attachment during mitosis.

### Ectopic WT 53BP1 but not the S1342A mutant, rescues 53BP1-depleted cells from merotely

**Figure 8:**
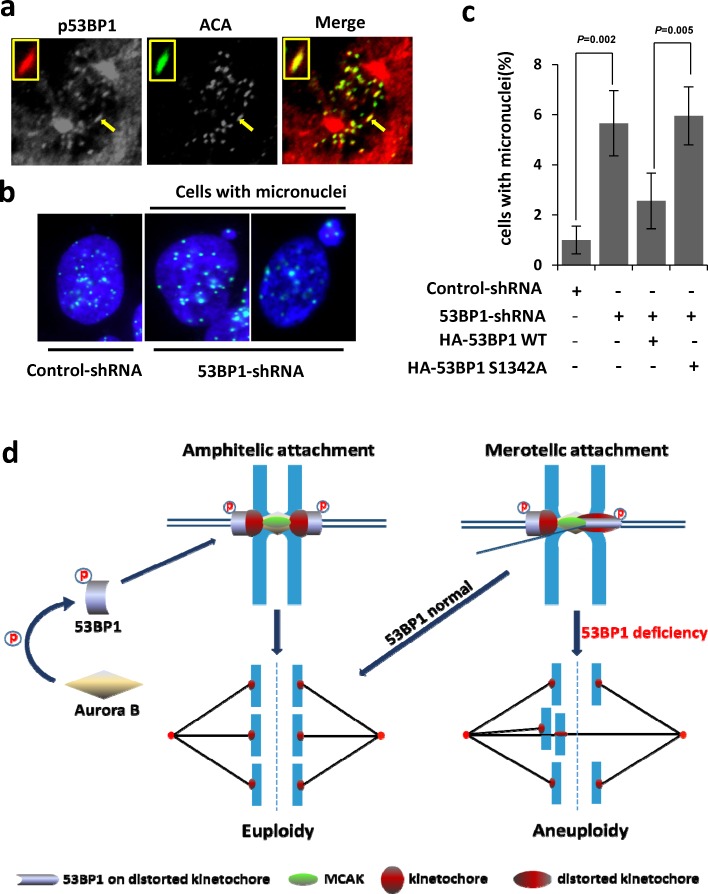
Ectopic WT 53BP1-KBD but not the S1342A mutant, rescues 53BP1-depleted cells from merotely **a**. Co-localization of phosphorylated (p)-S1342-53BP1with HEC1 (green) on distorted kinetochores (arrow). The inset shows magnified distorted kinetochores. **b**. Immunofluorescence showing increased formation of micronuclei containing kinetochores (ACA labeled in green) in 53BP1-depleted cells. **c**. Quantitation of micronuclei formation in (b), which is rescued by expression of ectopic HA-53BP1 WT 53BP1, but not by the HA-53BP1 S1342A mutant. Data are expressed as mean ± SD. **d**. Our model showing the critical role of Aurora B-dependent 53BP1 phosphorylation and optimal recruitment at kinetochores to protect against aneuploidy by merotely correction.

Following up our results showing increased merotely and aneuploidy in 53BP1-depleted cells (Figure 7), we examined the role of Aurora B-dependent 53BP1 phosphorylation in resolving spontaneous merotelic attachment errors. We then performed complementation analysis with ectopically expressed WT 53BP1 vs. the S1342A mutant in 53BP1-depleted U2OS cells. Expression of the WT protein but not S1342A mutant prevented aneuploidy in 53BP1-depleted cells as analyzed by quantitation of giemsa stained chromosomes (Figure 7c). Consistently, a strong co-localization of p-S1342 53BP1 was observed with distorted HEC1 staining on merotelic kinetochores (Figure 8a). Furthermore, the formation of micronuclei, small extranuclear bodies that are usually incorporated into merotelic kinetochore attachments induced by lagging chromosomes [39, 40], was analyzed. Again, ectopic WT, but not the S1342A mutant, prevented micronuclei formation in 53BP1-deficient cells (Figure 8b and 8c). Together, these data strongly suggest that Aurora B-mediated S1342-53BP1 phosphorylation is critical to guard human cells against spontaneous merotelic attachments that cause chromosome segregation errors during mitosis.

## DISCUSSION

In eukaryotic cells, accurate chromosome segregation is essential for preserving chromosomal fidelity in daughter cells and is therefore safeguarded by multiple, highly coordinated mitotic processes. Upon mitotic entry, kinetochores assemble on centromeres of duplicated chromatid pairs and each kinetochore is then captured by microtubule fibers from opposite spindle poles. This connection triggers chromosome alignment at the spindle equator and subsequently induces the onset of anaphase [41–46]. This highly regulated process has evolved with multiple factors to prevent, as well as to resolve, incorrect chromosomal segregation, failure of which has been implicated in tumorigenesis and therapeutic resistance [47–49].

Accurate attachment of kinetochores to the spindle during early mitosis is extremely critical but is intrinsically error prone [35, 50–52]. Multiple types of kinetochore-microtubule attachment errors can occur during this stage. Syntelic attachment is an error that both sister kinetochores are captured by microtubules from a single spindle pole, which activates the SAC [53] and induces mitotic arrest. A second type of error, namely merotelic attachment, spontaneously occurs when a single kinetochore of a centromere is connected by microtubules from both opposite spindle poles. Merotely spontaneously occurs during early mitosis but is normally resolved before anaphase begins while failure to resolve these errors prior to anaphase onset causes lagging chromosome formation and often leads to aneuploidy in human cells [19]. Previous studies have shown that Aurora B kinase re-orients merotelically-attached abnormal kinetochores and ensures timely correction of the merotely [16, 54]. However, the mechanism of merotelic attachment correction is poorly understood.

In this study, we identified 53BP1 as a novel component of the merotely correction pathway under the regulation of Aurora B via its phosphorylation at residue S1342. Sustained localization of p-S1342-53BP1 on merotelically attached kinetochores and rescue of micronuclei occurred in 53BP1-depleted cells by WT 53BP1, but not by the S1342A mutant, implicating S1342's specific phosphorylation in resolving these errors. While Aurora B predominantly regulates 53BP1 recruitment to the kinetochore, the participation of additional factors should be considered due to the few persistent staining of 53BP1 on kinetochores in cells treated with an Aurora B kinase inhibitor. In addition to Aurora B, MCAK protein also modulates 53BP1 activity. This kinesin is an important substrate of Aurora B involved regulating merotelic kinetochore-microtubule attachment [16]. Aurora B recruits MCAK to centromeres and merotelic attachments, and Aurora B then negatively modulates MCAK's depolymerization activity via its S196 phosphorylation. This step is required for importer microtubule release from merotelic attachments [33]. The kinesin-13 family includes three proteins (Kif2a Kif2b, and MCAK/Kif2c), all of which are involved with mitotic processing. Previous studies have shown that both Kif2b and MCAK are required for dynamic correction of merotelic attachments [55, 56]. We observed 53BP1's interaction with MCAK, but not with Kif2a or Kif2b (Supplementary Figure S5). The requirement of MCAK for 53BP1's distribution to merotelic kinetochores suggested a concerted pathway involving both 53BP1 and MCAK to cooperatively resolve merotely. While MCAK dynamically localizes throughout the inner centromere to the outer kinetochores during mitosis [57, 58], 53BP1's signal is extended from outer kinetochore to inner kinetochores that is merotelically attached, which appears to be highly regulated. The spatiotemporal interplay of 53BP1 and MCAK in maintaining chromosome fidelity warrants further investigation. In this work, we have demonstrated that 53BP1 is an essential component of the merotelic attachment correction cascade and is regulated by both Aurora B and MCAK. The critical role of Aurora B-dependent 53BP1 phosphorylation and optimal recruitment at kinetochores to protect against aneuploidy by merotely correction is schematically represented in Figure 8d.

53BP1 has established roles in DSB repair and DNA damage checkpoint activation. The lagging chromosome that we observed could be a consequence of DNA response defects. However, DNA damage-induced lagging chromosome is normally observed after exposure DSB-inducing agents like irradiation, which induce robust induction of DNA breaks, and in particular, when key cell checkpoint proteins are suppressed [59]. Our observations carried out in unstressed cells and within two-to three passages after transient 53BP1 depletion, ruled out a significant contribution from DNA damage. We are cognizant that endogenous DSBs could occur during cell cycle progression, however, their contribution, if any, should be minor because homologous recombination (HR) is unaffected (in fact, enhanced) after 53BP1 depletion [60]. In addition, 53BP1^−/−^ mouse model have shown that 53BP1 plays only a subtle role in intra-S-phase regulation and is not critical for G_1_ or early G_2_/M checkpoint control even after irradiation [4], which further supported the negligible impact of endogenous DSBs on lagging chromosome in 53BP1 depleted cells. Besides, the highly distorted kinetochore we observed in 53BP1 depleted anaphase cells is a phenomenon specifically caused by unresolved merotelic attachment due to driving force from opposite spindle poles, but not unrepaired DNA damage. Furthermore, rescue of this phenotype by 53BP1 WT but not S1342 mutant, led us to believe that 53BP1 plays a role in guarding against kinetochore-microtubule attachment errors during mitosis.

Finally, this study provides evidence that 53BP1 plays a critical role in maintaining faithful chromosome segregation in an Aurora B kinase activity-dependent manner. We identified an Aurora B consensus motif within the 53BP1's minimal KBD that contains the residue S1342, which is phosphorylated by Aurora B both *in vitro* and *in cellulo*. This phosphorylation is required for optimal 53BP1 binding to kinetochores. Importantly, our data demonstrate that 53BP1 ensures accurate chromosomal segregation by correcting merotelic attachments, a frequent error involving kinetochore-microtubule connections in mammalian cells. 53BP1 knockdown in human cells significantly enhanced merotelic attachments, as well as aneuploidy, thus underscoring the essential role of 53BP1 in maintaining chromosomal fidelity during mitosis and opens up new lines of investigation into its DNA damage-independent role in tumorigenesis. Many human cancers, including subsets of sporadic triple-negative and BRCA-associated breast cancer, are associated with reduced 53BP1 levels [7, 61]. Interestingly, it has been demonstrated that loss of 53BP1 rescues DNA repair defects in BRCA1 deficient cells [60], thus the dual loss of BRCA1 and 53BP1 in breast tumors is likely to contribute to cancer susceptibility via chromosome instability.

## MATERIALS AND METHODS

### Cell lines, antibodies, plasmids, and oligomers

Human osteosarcoma cell line U2OS and cervical cancer cell line HeLa were cultured in Dulbecco's modified Eagle's medium (Hyclone, Logan, UT), immortalized fibroblasts BJ hTERT was cultured in Dulbecco's modified Eagle's /F12 (Hyclone, Logan, UT) with 10% fetal bovine serum (Hyclone) and 100U/ml each of penicillin and streptomycin (Hyclone) and incubated at 37°C in the presence of 5% CO_2_. Rabbit polyclonal antibody against 53BP1 was purchased from Bethyl Laboratories, Inc. (Montgomery, TX). Anti-centromere antibody was obtained from Antibodies, Inc. (Davis, CA). Mouse antibodies against β-actin and FLAG-tag were purchased from Sigma-Aldrich (St Louis, MO), and rat anti-HA tag antibody was obtained from Roche (Basel, Switzerland). Mouse antibodies against Aurora B and HEC1 were obtained from Abcam (Cambridge, MA), and the rabbit MCAK antibody was a gift from Dr. D. A. Compton (Dartmouth Medical School). Two independent 53BP1 siRNA oligomers were purchased from Santa Cruz Biotechnology, Inc. (Dallas, TX) (pool of 3 different siRNA duplexes: 5′CCAUCAGUCAGGUCAUUGAtt, 5′GUGAACCAGUGGAGUUAGAtt and 5′CUCCAGUUCUCAAUUCUAAtt) and Dharmacon (Lafayette, CO) (5′GGACUCCAGUGUUGUCAUU), respectively. MCAK siRNA oligomers (pool of 3 different siRNA duplexes: 5′GGAAUGUCAUCCACUUACUtt, 5′GUACAUGAACCCAAGUUGAtt and 5′CUCUAGGACUUGCAUGAUUtt) were purchased from Santa Cruz Biotechnology, Inc., while control (Cat. SHC002) and 53BP1 shRNAs (Cat. SHCLNG-NM_005657) were purchased from Sigma-Aldrich. All fluorescent secondary antibodies including, Alexa Fluor 488 anti-mouse, -human and -rat antibodies, and Texas Red anti-rabbit antibody were obtained from Life Technologies (Grand Island, NY). The HA-Aurora B, GST-53BP1 (1300-1750), FLAG-53BP1, HA-53BP1- KBD were cloned using standard approaches. The S1341A mutant was generated by site-directed mutagenesis (Life Technologies). The primer sequences used for cloning are given in Supplementary Table 1. We custom-generated rabbit ployclonal p-S1342-53BP1 antibody using the services of EzBiolab (Carmel, IN), against a phosphopeptide sequence, LRGKT(pSer)GTEPADFAC. The antibody specificity was tested by immunoblotting with WT and phospho-peptide (Supplementary Figure S2c).

### Cell synchronization and drug treatment

Mitotic synchronization was performed by nocodazole (NOC) treatment (50 ng/ml for 8-14 h), and mitotic cells were collected by shake-off. Aurora B inhibitors ZM447439 and Hesperadin were purchased from Tocris Bioscience (Ellisville, MO) and EMD Millipore (Billerica, MA), respectively. Inhibitors ZM447439 or Hesperadin was added to the cells at a final concentration of 4 μM or 100 nM, respectively, followed by incubation for 4 h.

### Transfection, immunoblotting, co-IP and immunofluorescence (IF) assays

Plasmids and siRNA oligomers were transfected into U2OS or BJ hTERT cells by using the FuGENE HD (Promega, Madison, WI) or Lipofectamine 2000 (Invitrogen, Carlsbad, CA) and Oligofectamine (Life Technologies) systems, respectively, per the manufacturers' instructions. Immunoblotting, co-immunoprecipitation, and immunofluorescence were performed as previously described [62, 63]. Immunofluorescence images were captured for cells grown on cover slides (Olympus) or Nikon A1 confocal imaging system at 60X magnification. Immunoblotting images were cropped by Photoshop software.

### Time-lapse microscopy

U2OS cells stably expressing EGFP-H2B were transfected with control or 53BP1-siRNA/shRNA and inoculated into a 6-well plate. Images were captured with the IncuCyte live-cell imaging system (Essen Bioscience, Ann Arbor, MI) with 10-min intervals for 16 h. All images were analyzed with IncuCyte software.

### Protein purification and in vitro kinase activity assay

His-Aurora B was expressed and purified as previously described [64]. WT GST-53BP1 and mutant proteins were expressed in *E. coli* BL21 (DE3) strain (EMD Millipore) and affinity-purified on a Glutathione Sepharose column (GE Healthcare) as described previously [65, 66]. During the *in vitro* kinase assay, His-Aurora B was incubated with GST-53BP1 polypeptides at 30°C for 30 min in 1X kinase buffer containing 20 mM Tris [pH 7.4], 10 mM MgCl_2_, 0.5mM DTT, and 0.1 mM EDTA supplemented with 1mM ATP and 1μCi ^32^P-ATP [67].

### *In situ* proximity ligation assay (PLA)

The PLA assay was performed as previously described [8, 25]. Briefly, U2OS cells grown overnight in 4-well chamber slides were fixed with 4% paraformaldehyde and permeabilized with 0.2% Tween 20 followed by incubation with a primary antibody against 53BP1 (rabbit) or phosphorylated 53BP1 (rabbit) vs. Aurora B (mouse). We used the Duolink PLA kit from OLink Bioscience (Uppsala, Sweden) per the manufacturer's instructions. The nuclei were stained with DAPI, and the PLA signal was visualized with a fluorescence microscope at 60X magnification.

### Giemsa staining

Chromosome analysis at metaphase was done with and without colcemid treatment. Metaphases were prepared by standard procedures [68]. Chromosome spreads were prepared after hypotonic treatment of cells, fixed in acetic acid-methanol, and stained with Giemsa [69]. Giemsa-stained chromosomes from metaphase spreads were analyzed for chromosome numbers. Three hundred metaphases were analyzed and the data presented is the mean.

### Bioinformatics and statistical analysis

Sequence alignment was performed by BioEdit software; phosphorylation consensus motif was analyzed by software. Data were determined by Student's t test and *p* values < 0.05 were considered significant.

## SUPPLEMENTARY MATERIALS FIGURES AND TABLE


