# Supplementary material for: Aurora kinase B dependent phosphorylation of 53BP1 is required for resolving merotelic kinetochore-microtubule attachment errors during mitosis
Source: Oncotarget. 2017 Mar 15;8(30):48671–87. doi: 10.18632/oncotarget.16225 (PMC5564716; doi:10.18632/oncotarget.16225)
Supplement: Supplementary file 1 [file oncotarget-08-48671-s001.pdf]

Aurora kinase B dependent phosphorylation of 53BP1 is required for resolving merotelic kinetochore-microtubule attachment errors during mitosis

Supplementary Materials

Supplementary Table 1: Primer sequences used for cloning 53BP1 expression plasmids.

Primer Name	Sequence
h53BP1 KBD 3703 5'-Forward	5'-CCGCGTGAATTCGTCTTACATCGTCACATGAGAACAATCCGG
h53BP1 KBD 4848 5'-Reverse	5'-GCTCGATCTAGA ATCTGCTGCCTTTGTAAGAGGTGTTAC
h53BP1 S1342A F 5'-	5'-CCACTCAGAGGGAAAACCGCCGGGACAGAACCCGCAG
h53BP1 S1342A-R 5'	5'-CTGCGGGTTCTGTCCCGGCGGTTTTCCCTCTGAGTGG

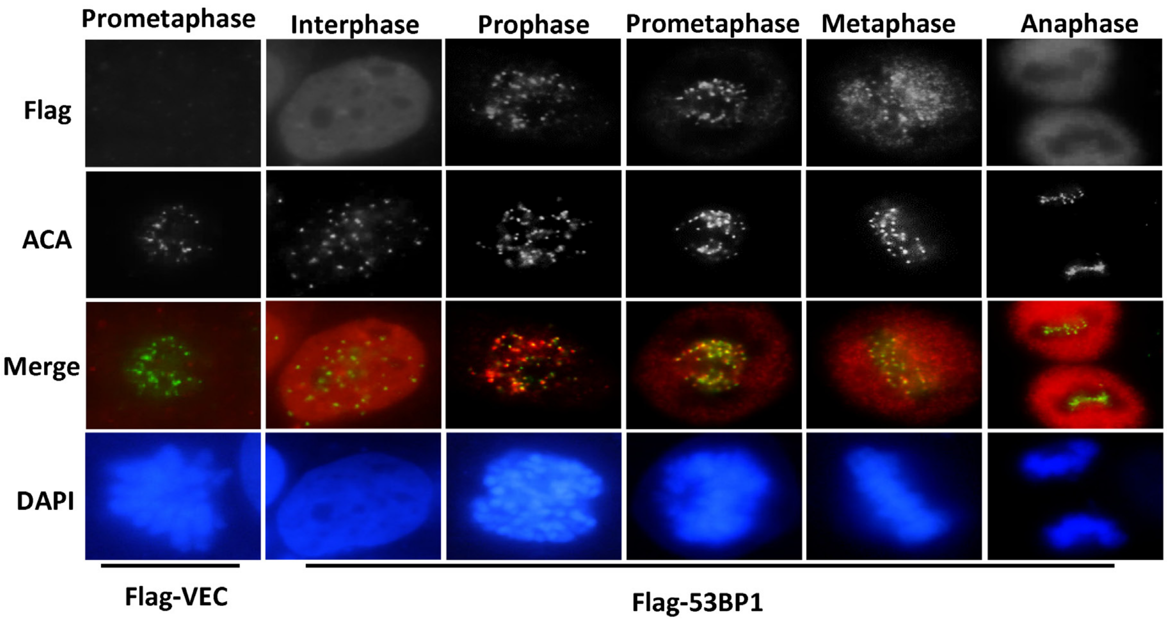

Supplementary Figure 1: Unsynchronized U2OS cells were transfected with FLAG-vector or FLAG-53BP1 plasmids and plated on cover slides. Images were captured 48 h post-transfection after immunostaining with anti-FLAG antibody (red) and ACA (green). Nuclei were indicated by DAPI (blue).

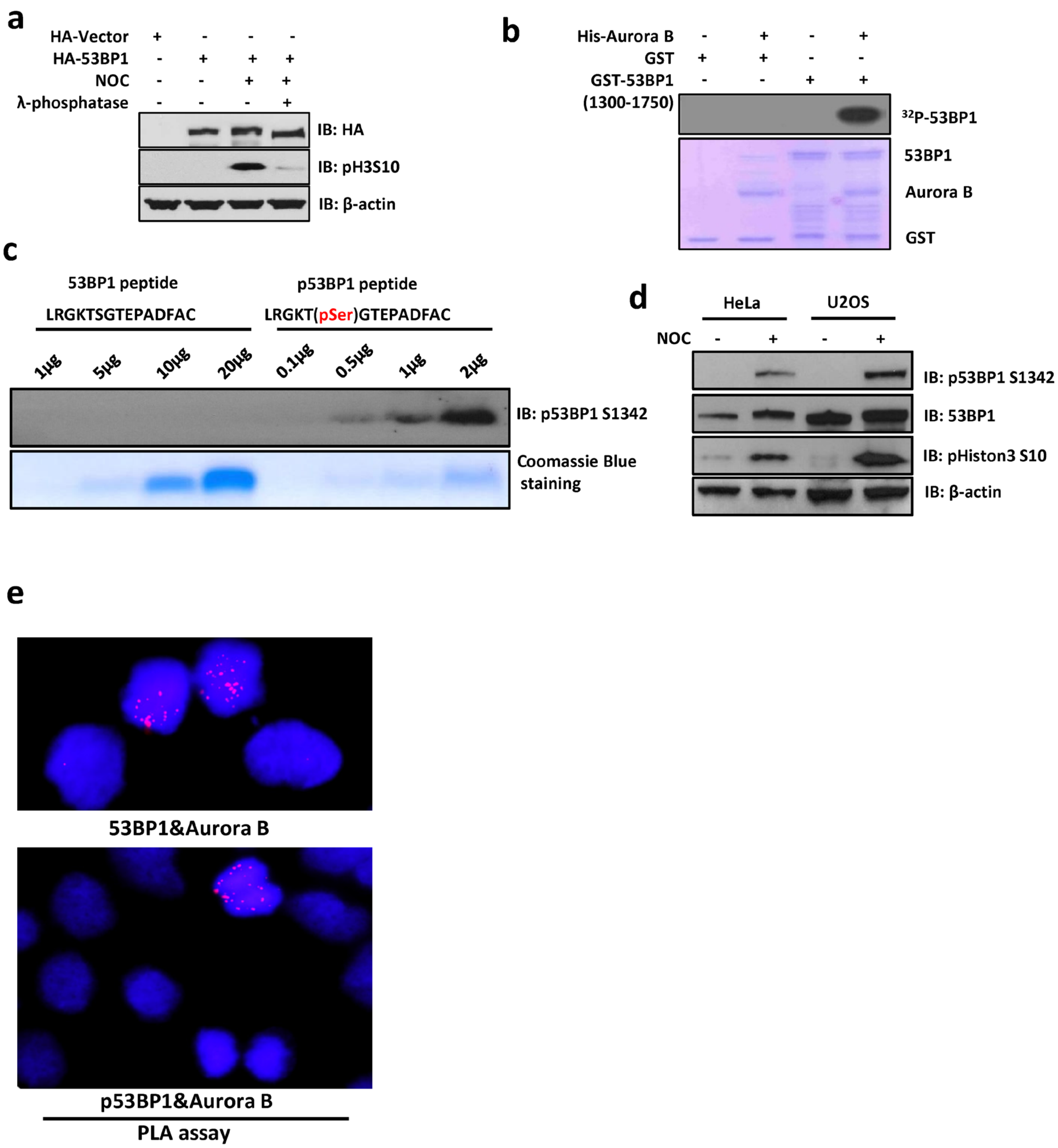

Supplementary Figure 2: (a) Western blotting shows that ectopically expressed Flag-53BP1 53BP1 is hyper-phosphorylated in NOC treated U2OS cells but dephosphorylated by treatment with λ-phosphatase**.** The phospho Histon3 S10 indicate mitotic arrest in response to NOC treatment. (b) An *in vitro* kinase assay revealed His-Aurora B phosphorylates GST-53BP1 (aa 1300-1750), but not GST alone. (c) Specificity of custom-generated p-S1342-53BP1 antibody with S1342 phosphorylated peptide was confirmed by Coomassie-stained SDS-PAGE with control and phosphorylated peptides (bottom). (d) Immunoblotting of mock- or NOC-treated HeLa and U2OS cell extracts. Mitotic cells were collected by shake-off. Phosphorylation of histone H3 at residue S10 is a mitotic marker. (e) An *in situ* proximity ligation assay shows interaction foci (red) of endogenous WT 53BP1 or p-S1342-53BP1 with Aurora B only in prometaphase cells.

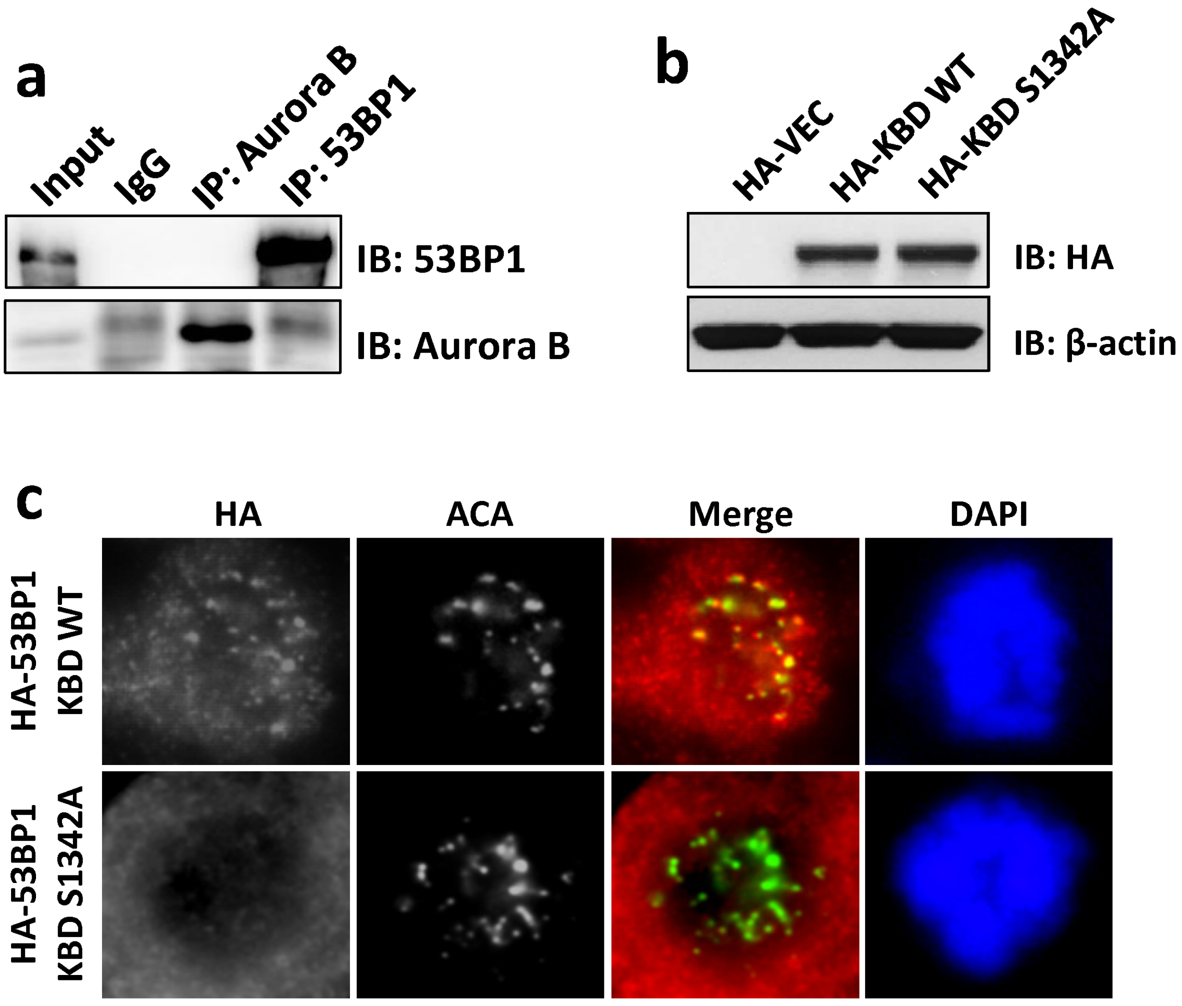

Supplementary Figure 3: (a) The interaction between 53BP1 and Aurora B was not detected in NOC synchronized U2OS cells by immunoblotting**.** (b) Immunoblotting shows that the expression of HA-KBD WT and HA-KBD S1342A in U2OS cells are comparable. (c) Immunostaining of HA (red) and ACA (green) in HA-53BP1 KBD WT and HA-53BP1 KBD S1342A transfected U2OS cells. Chromosomes were stained by DAPI.

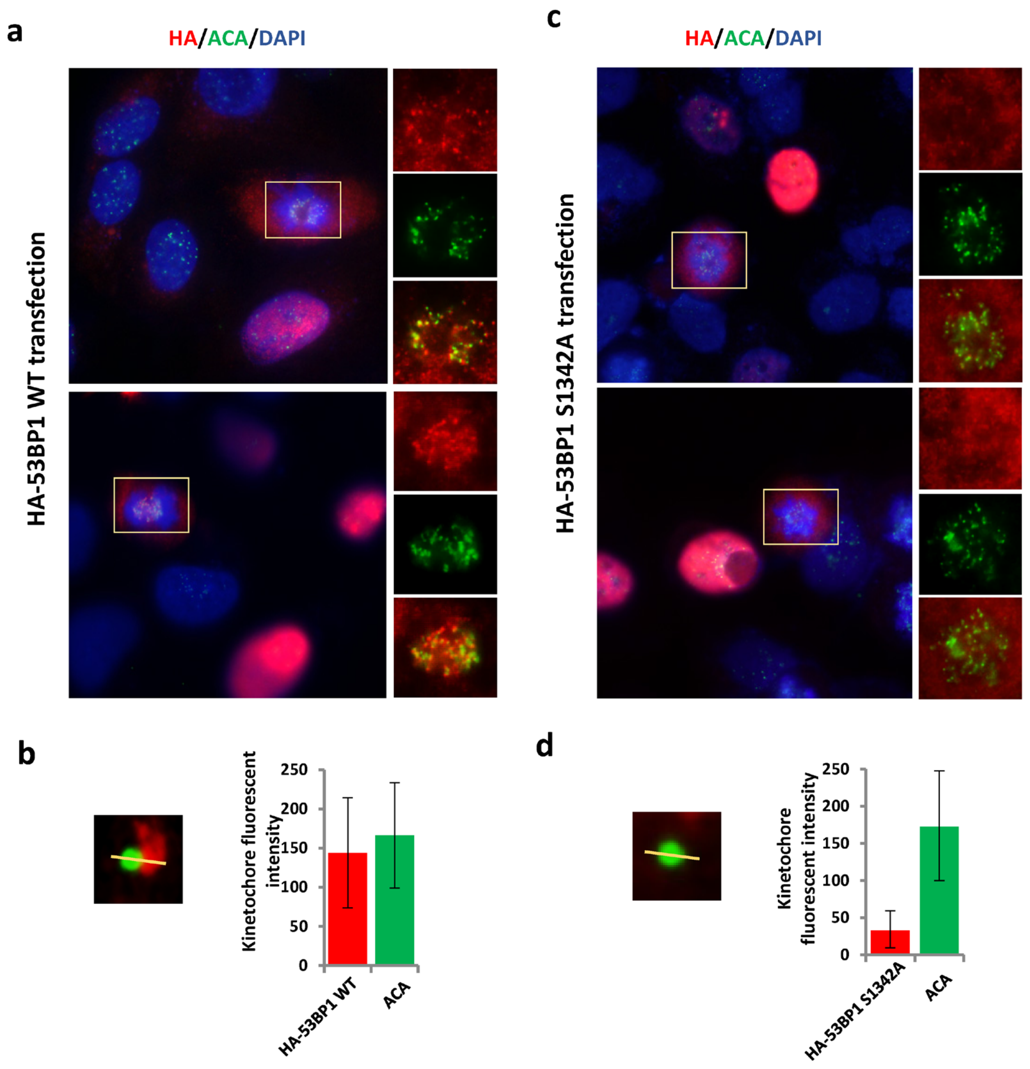

Supplementary Figure 4: Immunostaining of HA (red) and ACA (green) in HA-53BP1 WT (a) and HA-53BP1 S1342A (c) transfected U2OS cells. Chromosomes were stained by DAPI. (b and d)) Quantitation of fluorescent intensity of HA and ACA at kinetochores. 30 kinetochores were randomly selected and analyzed from four mitotic cells. Selected kinetochore staining was enlarged for visualization. The yellow line in images indicates that the fluorescent intensity was obtained by drawing of a 2-pixel-width line through the kinetochores.

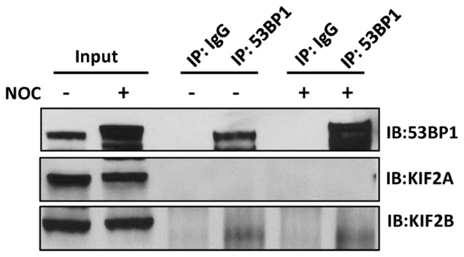

Supplementary Figure 5: Endogenous immunoprecipitation of 53BP1 from extracts of unsynchronized or NOC-synchronized mitotic cells. The interaction of 53BP1 with Kif2a or Kif2b is not observed.
